# Comprehensive Characterization of Long Non-Coding RNAs in Porcine Tissues: Expression Patterns and Functional Insights During Oocyte Development

**DOI:** 10.3390/cells14181409

**Published:** 2025-09-09

**Authors:** Yao Jiang, Yipeng Li, Qingpeng Shen, Xiaolong Yuan, Fei Gao, Bin Ma

**Affiliations:** 1 State Key Laboratory of Swine and Poultry Breeding Industry, Guangdong Laboratory of Lingnan Modern Agriculture, National Engineering Research Center for Breeding Swine Industry, Guangdong Provincial Key Lab of Agro-Animal Genomics and Molecular Breeding, College of Animal Science, South China Agricultural University, Guangzhou 510642, China; yao.jiang@murdoch.edu.au (Y.J.); liyipeng@stu.scau.edu.cn (Y.L.); qingpeng_shen@stu.scau.edu.cn (Q.S.); yxl@scau.edu.cn (X.Y.); 2 Centre for Healthy Ageing, Health Futures Institute, Murdoch University, Murdoch, WA 6150, Australia; 3 School of Medical, Molecular and Forensic Sciences, Murdoch University, Murdoch, WA 6150, Australia; 4 National Center of Technology Innovation for Pigs, Chongqing 402460, China; 5 Shenzhen Branch, Guangdong Laboratory for Lingnan Modern Agriculture, Genome Analysis Laboratory of the Ministry of Agriculture, Agricultural Genomics Institute at Shenzhen, Chinese Academy of Agricultural Sciences, Shenzhen 518000, China

**Keywords:** long non-coding RNAs (lncRNAs), pig, RNA-sequencing, oocyte development, functional enrichment, tissue-specific expression

## Abstract

Long non-coding RNAs (lncRNAs) are essential regulatory molecules involved in various biological processes in mammals. However, their expression patterns across multiple porcine tissues have not been systematically characterized. We analyzed 607 RNA-seq datasets derived from 14 porcine tissues, including backfat, gallbladder, heart, ileum, jejunum, kidney, longissimus dorsi, liver, lung, skeletal muscle, ovary, pituitary, skeletal muscle, and spleen. Additionally, we examined 63 single-cell RNA-seq datasets from porcine primary oocytes at five developmental stages. For comparative analysis, we included 20 human and 17 mouse oocyte RNA-seq datasets. We identified 52,798 porcine lncRNAs, with tissue-specific expression patterns most prominent in oocytes and least in skeletal muscle. Among them, 2169 were classified as housekeeping and 14,469 as tissue-specific lncRNAs. Cross-species analysis revealed that a small subset of oocyte-expressed lncRNAs is conserved in humans and mice, associated with catalytic activity and circadian regulation. Additionally, 44 lncRNAs were differentially expressed during oocyte development, implicating them in neurogenesis, vesicle transport, and protein modification. Our findings not only contribute to the growing body of knowledge regarding lncRNAs in porcine biology but also pave the way for future research aimed at elucidating their functional roles in reproductive biology and other physiological processes.

## 1. Introduction

Pigs are significant economic mammals due to their efficient conversion of feed to meat, high reproductive rates, and the wide range of uses for various parts of the animal. They are a primary source of meat for many families and are also used for leather, fat, and other byproducts in various industries [1]. Understanding the mechanisms involved in growth and reproduction is crucial for improving breeding/production strategies, enhancing meat quality, and optimizing growth rates in livestock [2,3]. In addition, genetics and genetic editing in pig breeding are critical areas of research that aim to enhance desirable traits, improve health, and increase productivity in swine [4]. Except as economic animals, pigs are valuable models for human disease research because of their physiological and genetic similarities to humans [5,6]. Their organ systems and metabolic processes closely resemble those of humans, making them ideal for studying complex diseases such as diabetes, cardiovascular conditions, and obesity. Additionally, pigs can be genetically modified to create models that mimic specific human diseases, allowing for more targeted research. This similarity enhances the translational potential of findings from pig studies to human applications, facilitating the development of new therapies and interventions [7].

Long non-coding RNAs (lncRNAs) are a class of RNA molecules, typically defined as transcripts longer than 200 nucleotides (nt) that are not translated into proteins [8]. These lncRNAs can interact with DNA, mRNA, microRNAs, and proteins, thereby influencing key biological processes such as chromatin remodeling, transcription, and post-transcriptional regulation [9]. As epigenetic regulators, lncRNAs play vital roles in modulating gene expression, cell differentiation/apoptosis, and growth/developmental processes [10]. Moreover, lncRNAs are significantly implicated in the development of various diseases, particularly cancer, cardiovascular diseases, and neurological disorders. For instance, altered expression levels of specific lncRNAs have been associated with several neurodegenerative, neurodevelopmental, and mental health disorders [11]. Understanding the mechanisms through which lncRNAs contribute to these diseases may pave the way for novel therapeutic targets and biomarkers, offering new avenues for treatment and diagnosis [12,13].

The mechanisms through which lncRNAs function are intricate and not yet fully elucidated. Based on their cellular localization, lncRNAs can be categorized into two main types: cytoplasmic lncRNAs and nuclear lncRNAs. Nuclear lncRNAs are often considered the dominant type due to their significant roles in regulating gene expression, chromatin structure, and transcriptional processes within the nucleus. They interact with chromosomes, chromatin domains, and specific genomic loci, influencing cellular functions and contributing to the complexity of gene regulation and nuclear function [14]. In contrast, cytoplasmic lncRNAs exhibit a remarkable capacity to interact with various cellular structures (including ribosomes, the cytoskeleton, the endoplasmic reticulum, and mitochondria), allowing them to influence mRNA transport, stability, and translation [8,15]. Additionally, cytoplasmic lncRNAs play essential roles in modulating protein stability, post-translational modifications, and functional activities [16]. The mechanistic diversity of cytoplasmic lncRNAs encompasses not only the regulation of cellular architecture but also the direct control of protein synthesis and function, highlighting their multifaceted contributions to cellular homeostasis.

lncRNAs exhibit differential expression across various tissues, with certain lncRNAs being abundant in specific tissues while either absent or present at lower levels in others [17,18]. For instance, some lncRNAs are predominantly expressed in the brain, influencing neuronal development and function, while others are enriched in muscle tissue, playing critical roles in muscle differentiation and regeneration [19,20]. Research has demonstrated that lncRNAs are pivotal in regulating essential economic traits such as muscle development and fat deposition, primarily through mechanisms involving epigenetic regulation and chromatin remodeling [21,22].

Transcriptomic analyses of the longissimus dorsi muscle have unveiled a regulatory network of lncRNAs involved in muscle fiber differentiation [23]. Additionally, studies indicate that lncRNAs significantly influence fat deposition in the adipose tissue of Ningxiang pigs, particularly through the sphingolipid metabolic pathway [24]. In the realm of reproductive traits, the expression of lncRNAs in porcine oocytes is closely associated with DNA methylation modifications [25]. However, most existing research has focused on individual tissues, with limited exploration of lncRNAs within multi-tissue regulatory systems, such as the hypothalamic-pituitary-ovarian axis. The dynamic expression patterns of lncRNAs across multiple tissues and their conservation across species during oocyte development remain underexplored. This gap in systematic investigation constrains the potential applications of lncRNAs in pig breeding programs and other biomedical research utilizing pig as a disease model.

In contrast to lncRNAs, short non-coding RNAs, particularly microRNAs, display a higher degree of conservation across species due to their fundamental roles in gene regulation and cellular processes [26,27]. The lack of conservation of lncRNAs compared to miRNAs and mRNAs is often linked to their diverse regulatory roles in biological processes [8,28]. lncRNAs may not be conserved across species due to rapid evolution, functional redundancy, tissue-specific expression, changes in genomic context, and functional divergence. These factors allow lncRNAs to adapt to unique regulatory roles in different species, leading to significant sequence variability and a lack of conservation [29]. Despite the significance of lncRNAs, there is a notable scarcity of studies investigating their cross-species conservation, which limits our understanding of their evolutionary significance and functional implications. Further research is crucial to elucidate the conservation patterns of lncRNAs and their impact on regulatory networks across species.

In this study, we aim to address the gaps in our understanding of tissue-specific and species-specific expression patterns of lncRNAs by leveraging publicly available transcriptomic datasets in conjunction with our own generated datasets. Furthermore, we seek to clarify the regulatory mechanisms, networks, and pathways associated with these lncRNAs.

## 2. Materials and Methods

### 2.1. Sample/Data Collection

A total of 607 RNA-seq datasets were obtained from the Sequence Read Archive (SRA; https://www.ncbi.nlm.nih.gov/sra, accessed on 22 September 2022), encompassing 14 different tissues from pigs (Supplemental Table S1). The tissue distribution included backfat (*n* = 31), gallbladder (*n* = 19), heart (*n* = 28), ileum (*n* = 28), jejunum (*n* = 21), kidney (*n* = 16), longissimus dorsi muscle (*n* = 96), liver (*n* = 64), lung (*n* = 77), muscle (*n* = 28), ovary (*n* = 55), pituitary (*n* = 26), muscle (*n* = 72, including longissimus muscle and skeletal muscle), and spleen (*n* = 46). Additionally, 53 porcine oocyte samples were collected from four healthy Large White × Landrace crossbred gilts (approximately 210 days old) that exhibited signs of estrus. To investigate the alterations in the expression patterns of long non-coding RNAs (lncRNAs) throughout follicular development, antral follicles (AF) were categorized into five distinct stages according to their diameters. These stages are designated as follows: AF1 (0.5–1.8 mm), AF2 (1.8–3 mm), AF3 (3–5 mm), AF4 (5–7 mm), and AF5 (>7 mm), and the transcriptomes of oocytes from these AFs were conducted on the single-cell RNA-seq [30].

Human oocyte RNA-seq data were retrieved from the Gene Expression Omnibus (GEO) under accession number GSE107746, which included 20 samples. Mouse oocyte RNA-seq datasets were sourced from GEO accession numbers GSE141190, GSE70116, GSE116771, GSE74344, and GSE73803, comprising a total of 17 samples for analysis.

### 2.2. Quality Control, Read Alignment, and Transcriptome Assembly

The samples were transformed into paired-end FASTQ format using the fast-dump module of the SRA Toolkit (version 2.8.2). The quality of the raw sequencing data was assessed using FASTP software (version 0.23.2) with default parameters, which allowed for filtering and trimming of the reads to eliminate low-quality bases and adaptors [31]. Following this, the clean data were mapped to the reference genome using HISAT2 software (version 2.1.0), and the resulting SAM files were converted to BAM format using Samtools (version v1.11).

RNA-seq data from each sample were processed with StringTie to generate transcript-level GTF files. These files were subsequently merged using StringTie’s merge function [32], resulting in a unified GTF that includes all predicted transcripts. Transcript annotation was performed using gffcompare [33], which identifies relationships between predicted and reference transcripts, such as antisense overlap, intronic location, and shared splice sites.

Transcripts were retained based on the following criteria: (i) antisense overlap with a reference transcript; (ii) being entirely intronic; (iii) sharing splice sites; (iv) exon overlap; (v) being located in intergenic regions; or (vi) sharing intron boundaries. The filtered GTF was then utilized with gffread to extract transcript sequences in FASTA format.

### 2.3. lncRNA Detection

Candidate sequences were evaluated for coding potential using three tools: CNCI [34], CPC2, and FEELnc [35]. Transcripts were classified as lncRNAs if they scored less than 0 in CNCI, less than or equal to 0 in CPC2, or fell below the machine-learning-derived threshold in FEELnc. Only sequences consistently identified as non-coding by these tools were retained for further analysis.

### 2.4. Descriptive Statistics of lncRNAs and Expression Quantification

We conducted descriptive analyses of lncRNAs expressed in each tissue, focusing on statistics related to their number, length distribution, and classification based on genomic location. According to the annotations provided by FEELnc, lncRNAs were categorized into four types based on their genomic context: exonic, intronic, upstream, and downstream. We then calculated the proportion of expressed lncRNAs in each category for every sample. Statistical differences in lncRNA positional distribution among tissues were determined using *t*-test.

Expression quantification of lncRNAs was performed using featureCounts [36], resulting in a count matrix for downstream analysis. To minimize false positives, we retained only those lncRNAs with more than 10 reads in at least three samples of a single tissue type, ensuring robust and reproducible expression results.

### 2.5. t-SNE Clustering Analysis of lncRNAs

t-distributed stochastic neighbor embedding (t-SNE) was utilized for dimensionality reduction and clustering analysis to investigate the expression pattern relationships of lncRNAs across different samples [37]. To further assess the correlation between tissues, Spearman′s rank correlation coefficient was calculated [38].

### 2.6. Identification of Housekeeping and Tissue-Specific lncRNAs

Housekeeping lncRNAs were identified based on two criteria: (i) the average expression level of the lncRNA across all tissues exceeded the median expression level of all genes, and (ii) the coefficient of variation (CV) of its expression across tissues was less than 1. This approach allowed for the identification of lncRNAs that may play regulatory roles throughout the organism. These lncRNAs exhibit relatively high and stable expression across multiple tissues and are believed to be crucial in various biological processes.

Tissue-specific lncRNAs were identified based on the following conditions: (i) their expression level in a given tissue ranked within the top 25% of all lncRNAs; (ii) their expression in that tissue accounted for more than 50% of their total expression across all tissues; and (iii) their expression in that tissue was at least three times higher than in any other tissue. Such lncRNAs are likely involved in tissue-specific physiological or developmental processes.

To further explore the relationship between lncRNAs and mRNAs, a similar methodology was employed to identify housekeeping and tissue-specific mRNAs.

### 2.7. Identification of mRNAs and circRNAs in Oocytes

Sequence alignment was initially conducted using HISAT2 (version 2.1.0), followed by transcript reconstruction and expression estimation with StringTie (version 2.1.5). Gene- and transcript-level read counts were subsequently obtained using featureCounts (version 2.0.1). Utilizing previously established criteria for identifying housekeeping and tissue-specific mRNAs [39], we successfully identified both categories within the oocyte transcriptome.

To further investigate the interactions between circular RNAs (circRNAs) and lncRNAs in oocytes, we systematically identified circRNAs from 55 oocyte samples. The same quality control and alignment procedures applied to lncRNAs were used to ensure consistency and comparability across datasets. CircRNAs were accurately detected using CIRI2 software (version 2.0.6) [40], and their expression patterns and characteristics were analyzed to explore their potential interactions with lncRNAs in oocytes.

### 2.8. Construction of the ceRNA Network

To investigate the potential regulatory interactions among tissue-specific lncRNAs, miRNAs, and mRNAs, we constructed a competing endogenous RNA (ceRNA) network centered on tissue-specific lncRNAs. Here, we define the mRNAs predicted to interact with miRNAs in the lncRNA–miRNA–mRNA axis as the downstream target mRNAs of lncRNAs. lncRNA–miRNA and miRNA–mRNA interactions were predicted using miRanda (version 3.3a) with parameters score > 140 and energy < −20 kcal/mol. Only interactions supported by miRBase-annotated miRNA binding sites were retained. The circRNA–miRNA interactions were identified using the same procedure. Cytoscape (version 3.10.0) was used to integrate these interactions into a circRNA/lncRNA–miRNA–mRNA network.

Initially, sequence information for lncRNAs and mRNAs was extracted using BEDTools2 (version 2.23.0), while miRNA binding site sequences were sourced from miRBase (version v22.1) [41]. miRanda was then employed to predict interactions between miRNAs and lncRNAs, as well as between miRNAs and mRNAs. Downstream target mRNAs were defined as protein-coding transcripts whose 3′ UTRs were predicted to bind miRNAs that also interacted with the identified lncRNAs or circRNAs, thereby representing potential ceRNA relationships in which lncRNAs/circRNAs act as molecular sponges for shared miRNAs. By evaluating binding scores and minimum free energy, we assessed the stability and potential strength of these interactions. Finally, to visualize the regulatory network, we selected the top five lncRNAs with the highest interaction scores and mapped their ceRNA relationships using Cytoscape software [42].

### 2.9. Functional Enrichment Analysis of lncRNA-Associated Genes

In general, genes located within ±100 kb of the lncRNA locus and exhibiting a correlation coefficient greater than 0.6 with the lncRNA are considered as its potential cis-regulatory target genes. Using the annotation results from FEELnc, we identified the nearest potential parental protein-coding genes for each lncRNA to investigate their possible cis-regulatory roles. These genes were subsequently subjected to Gene Ontology (GO) and Kyoto Encyclopedia of Genes and Genomes (KEGG) enrichment analyses to explore the functional regulatory network associated with lncRNAs.

GO enrichment analysis was conducted using the clusterProfiler R package (version 3.14) and visualized with ggplot2 (version 3.3.5) [17], which revealed significant enrichment of lncRNA-associated genes across cellular components, biological processes, and molecular functions. For KEGG pathway enrichment analysis, we employed the ShinyGO (version 0.7.6) tool to identify overrepresented signaling and metabolic pathways [43]. In both analyses, statistical significance was assessed using the hypergeometric test, with a *p*-value threshold of <0.05 deemed significant.

### 2.10. Co-Expression Network Analysis

We employed Weighted Gene Co-expression Network Analysis (WGCNA) to investigate the co-expression patterns of lncRNAs across various tissues [44]. The analysis was conducted using the WGCNA package in R (version 1.70). WGCNA-based clustering was performed using the average linkage method (hclust function), and the soft-thresholding power (β) was determined with the “pickSoftThreshold” function in WGCNA, which defines the strength of connections between lncRNAs.

We focused on modules with a correlation coefficient greater than 0.65, which were considered to exhibit significant tissue specificity in this study. After selecting an appropriate soft-thresholding power, we constructed the co-expression network and identified key tissue-specific modules using WGCNA. Additionally, we identified critical tissue-specific modules from these co-expression networks and utilized module membership (MM) and gene significance (GS) to determine the core lncRNAs, referred to as hub lncRNAs. Hub lncRNAs were defined based on pre-established thresholds, typically |GS| > 0.2 and |MM| > 0.8, indicating strong correlations between lncRNAs and the module characteristic genes.

### 2.11. Inter-Species Conservation Analysis of lncRNAs

To facilitate the conversion of genomic positions, we first downloaded the necessary files for genome conversion from the UCSC Genome Browser: hg38ToSusScr11.over.chain.gz and mm39ToSusScr11.over.chain.gz. These files contain the mapping relationships from the human (hg38) and mouse (mm39) genomes to the pig genome (susScr11).

Utilizing the Liftover tool from the UCSC Genome Browser [45], we mapped the lncRNAs identified in the human (hg38) and mouse (mm39) genomes to their corresponding positions in the pig genome (susScr11), applying a parameter of “minMatch = 0.5” to ensure a minimum match rate.

Subsequently, we compared the resulting pig-genome-aligned lncRNAs with the lncRNA positions obtained from 15 different pig tissues. By examining the overlap of genomic positions, we identified lncRNAs that are conserved across species, which we refer to as inter-species conserved lncRNAs.

Short Time-series Expression Miner (STEM v1.3.13) was used with the following parameters: maximum model profiles = 50, minimum correlation coefficient = 0.7. Significant clusters were determined using Bonferroni-adjusted *p* < 0.05.

### 2.12. Statistical Analysis

Spearman′s correlation coefficient was utilized to compare the expression levels of lncRNAs across different tissues and time points for significant differences. In the context of descriptive statistics across multiple tissues, *t*-tests were employed to detect differences between tissue types. For GO analysis using the clusterProfiler R package (version 3.14) and KEGG analysis using ShinyGO (version 0.76), a significance threshold of *p* < 0.05 was applied. Additionally, Pearson′s correlation coefficient was used for correlation analyses among lncRNAs, mRNAs, and circRNAs.

Differential expression analysis of lncRNAs was performed in R using DESeq2 (v1.30.1). Raw counts from featureCounts were normalized by the median-of-ratios method. For pairwise comparisons among the five oocyte stages (AF1–AF5), a negative binomial Wald test was applied, and *p* values were adjusted using the Benjamini–Hochberg procedure. lncRNAs with an adjusted *p* value (FDR) < 0.05 and an absolute log2 fold change ≥ 1 were considered differentially expressed. Low-abundance lncRNAs were filtered prior to testing (read count > 10 in ≥ 3 samples of at least one stage/tissue, consistent with Section 2.4).

### 2.13. Molecular Validation

To ensure the accuracy of the identified lncRNAs, we randomly selected four housekeeping lncRNAs, known for their stable expression across all cell types, for experimental validation. Validation was performed using spleen tissue, chosen due to its stable RNA quality and availability. We employed polymerase chain reaction (PCR) to amplify and confirm the presence of these four lncRNAs.

Total RNA was isolated from freshly dissected spleen tissues using TRIzol reagent following the manufacturer’s instructions. Genomic DNA was removed by on-column DNase I treatment (10 U, 15 min) to avoid DNA contamination. RNA concentration and purity were assessed by spectrophotometry (A260/280 between 1.9 and 2.1; A260/230 > 2.0), and integrity was evaluated by agarose gel electrophoresis. Only samples meeting these criteria were used for downstream analyses. The validation process involved several steps: First, the sequences of the housekeeping lncRNAs were extracted based on their known genomic locations, and specific primers were designed for each lncRNA (Supplemental Table S2). After the primers were designed, the PCR mixture was prepared, following established PCR protocols while making necessary adjustments to suit the experimental requirements (Supplemental Table S3). Once the PCR mixture was thoroughly mixed, it was transferred to a PCR thermal cycler, where temperature cycling was applied according to the specified program settings (Supplemental Table S4). Finally, the PCR products were analyzed using gel electrophoresis, which allowed for the separation and visualization of the target fragments.

## 3. Results

### 3.1. lncRNA Expression Profiles in 15 Porcine Tissues

In this study, we analyzed a total of 607 samples derived from 14 different tissues and 53 oocyte samples, resulting in the generation of 3,700,474 transcripts. These transcripts were aligned to the reference genome annotation using GffCompare, leading to the retention of 3,053,113 transcripts for subsequent lncRNA identification.

**Figure 1:**
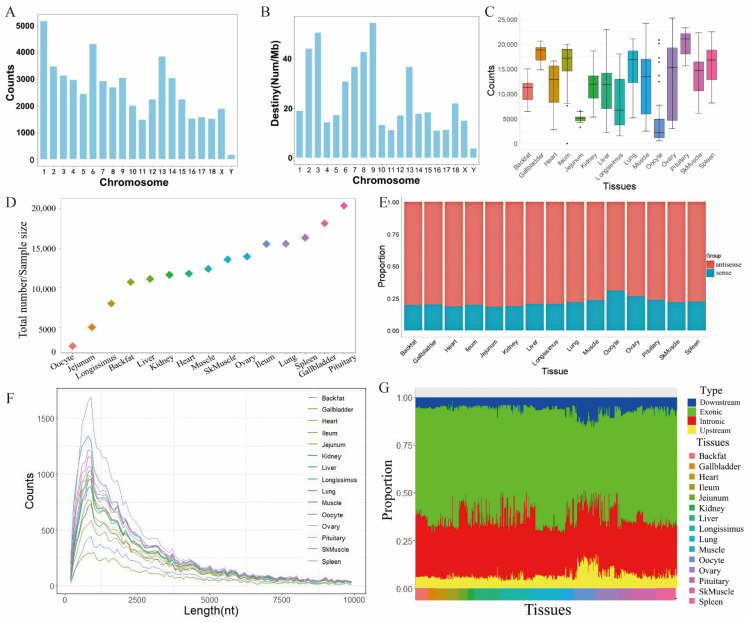
Genomic Distribution and Expression Patterns of lncRNAs Across 15 Pig Tissues. (**A**) Total number of lncRNAs identified across 607 samples from 15 tissues. lncRNAs were most abundant on Chromosome 1 and least abundant on Chromosome Y. (**B**) Density distribution of lncRNAs, with the highest density on Chromosome 9 and the lowest on Chromosome Y. (**C**) Boxplot of lncRNA expression per sample across tissues, revealing the highest expression in the pituitary gland (20,360) and the lowest in oocytes (2693). Black dots represent outliers, which are data points beyond 1.5 times the interquartile range (IQR) from the box, indicating individual samples with relatively higher or lower expression levels. (**D**) Scatter plot of lncRNA expression across 15 tissues. (**E**) Strand distribution of lncRNAs, with ~25% on the sense strand and ~75% on the antisense strand. Oocytes had a significantly lower proportion on the antisense strand (*p* = 0.023). (**F**) Length distribution of lncRNAs, peaking at 800–900 nt across tissues. (**G**) Genomic location distribution, with the majority found in exonic regions (43.25–61.95%). Oocytes had a lower exonic proportion (43.25%, *p* = 0.048) and higher upstream (13.35%, *p* = 0.016) and downstream (11.65%, *p* = 0.016) proportions.

To predict lncRNAs, three bioinformatics tools, namely CNCI, CPC2, and FEELnc were employed. These tools identified 255,693, 242,318, and 325,420 candidate lncRNAs, respectively. The intersection of the results from these three tools yielded a total of 72,931 candidate lncRNAs. To minimize false positives, stringent quality control measures were applied, ultimately confirming 52,798 lncRNAs. Our analysis of chromosomal distribution revealed that lncRNAs were most abundant on Chromosome 1 and least abundant on Chromosome Y. Additionally, lncRNA density was highest on Chromosome 9 and lowest on Chromosome Y (Figure 1A,B).

To investigate the differential expression of lncRNAs across various tissues, we first quantified lncRNA expression in all samples from the 15 tissues studied. We then determined the number of expressed lncRNAs in each tissue based on sample size. The results indicated that among the 15 tissues, lncRNAs were most abundantly expressed in the pituitary gland, with 20,360 lncRNAs detected. In contrast, oocytes exhibited the lowest number of expressed lncRNAs, with only 2693 identified (Figure 1C,D).

To explore the relationship between strand orientation and lncRNA function, we analyzed the strand distribution of lncRNAs across each tissue. The findings revealed that approximately 25% of lncRNAs were located on the sense strand, while 75% were found on the antisense strand (Figure 1E). However, the strand distribution varied significantly among different tissues, with oocytes showing a notably lower proportion of lncRNAs on the antisense strand compared to other tissues (*p* = 0.023).

We also conducted a statistical analysis of lncRNA length distribution, which demonstrated a consistent pattern across the 15 tissues. As lncRNA length increased, the number of lncRNAs gradually rose, peaking in the 800–900 nt range. Beyond this length, the number of lncRNAs began to decline, indicating that the length distribution is consistent across different tissues (Figure 1F).

To further explore the positional distribution of lncRNAs, we performed a statistical analysis of their genomic locations across all tissues (Figure 1G). The results showed that lncRNAs were distributed among four genomic regions: upstream, downstream, exonic, and intronic regions. The majority of lncRNAs were found in exonic regions, ranging from 43.25% in oocytes to 61.95% in the lung. The second most common location was the intronic region, with lncRNAs comprising 25.95% in the ileum and 33.15% in the longissimus dorsi muscle. Conversely, fewer lncRNAs were located in upstream regions (4.90% in the heart to 13.35% in oocytes) and downstream regions (4.20% in the jejunum to 11.65% in oocytes).

Notably, the positional distribution of lncRNAs in oocytes differed significantly from that in other tissues (*p* < 0.05). Compared to other tissues, oocytes exhibited a lower proportion of lncRNAs in exonic regions (43.25%, *p* = 0.048) but a higher proportion in upstream (13.35%, *p* = 0.016) and downstream regions (11.65%, *p* = 0.016). These findings suggest that lncRNA distribution in oocytes is distinctly different from that in other tissues.

### 3.2. Expression Characteristics and Functional Insights of lncRNAs Across Porcine Tissues

**Figure 2:**
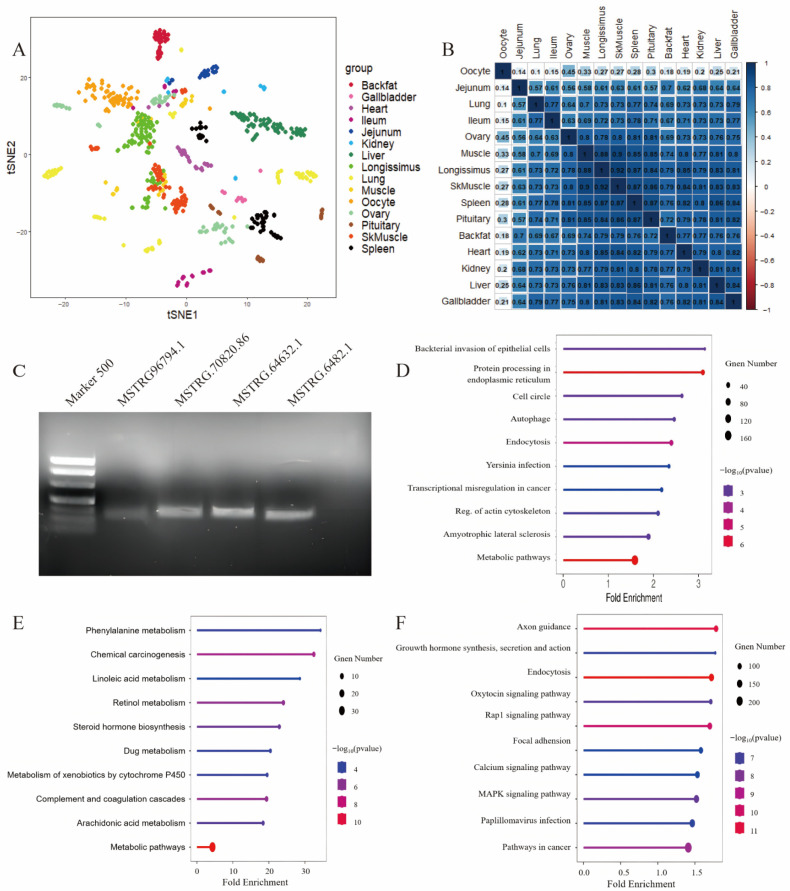
Expression characteristics and functional insights of lncRNAs across porcine tissues. (**A**) t-SNE clustering of lncRNA expression in 15 porcine tissues. The first character in the sample name represents the individual, the second indicates the developmental stage, and the third reflects the replicate number. (**B**) Spearman correlation analysis of lncRNA expression across the 15 tissues. (**C**) Gel electrophoresis results validating the expression of housekeeping lncRNAs in porcine spleen. (**D**) KEGG enrichment analysis of cis-regulatory target genes of housekeeping lncRNAs, revealing their involvement in protein processing, signal transduction, and cell cycle pathways. (**E**) KEGG enrichment analysis of liver-specific lncRNAs, showing enrichment in metabolic pathways including phenylalanine metabolism, linoleic acid metabolism, and retinol metabolism. (**F**) KEGG enrichment analysis of oocyte-specific lncRNAs, suggesting their involvement in cell morphology regulation and developmental transitions.

We conducted a thorough investigation into the variation in lncRNA expression patterns across different tissues using t-SNE clustering. This analysis revealed distinct t-SNE clustering in seven out of the 15 tissues examined: back fat, heart, jejunum, ileum, kidney, lung, and oocytes (Figure 2A). These results suggest a strong tissue-specific expression of lncRNAs and their potential roles in tissue-specific biological functions.

To further assess the relationships among tissues based on lncRNA expression, we calculated pairwise Spearman correlation coefficients (Figure 2B). Tissues with similar physiological roles, such as skeletal muscle and longissimus dorsi, exhibited high correlation coefficients (r = 0.88–0.92), indicating comparable regulatory profiles. In contrast, oocytes displayed significantly lower correlation with other tissues (r = 0.1–0.45), underscoring their unique transcriptional landscape and specialized regulatory roles.

To confirm the presence of the identified IncRNAs at the molecular level, we extracted total RNAs from porcine spleen and reverse-transcribed them into complementary DNAs (cDNAs). Four randomly selected IncRNA sequences were subjected to PCR amplification, and the results validated their transcriptional expression in pigs (Figure 2C).

To explore the functions of housekeeping lncRNAs, we first identified their cis-regulatory target genes and performed Kyoto Encyclopedia of Genes and Genomes (KEGG) enrichment analyses. The KEGG enrichment analysis indicated that these genes are critically involved in intracellular protein processing, signal transduction, and cell cycle regulation (Figure 2D). These findings suggest that housekeeping lncRNAs may play a significant role in complex regulatory mechanisms essential for maintaining cellular homeostasis and overall biological functions.

In liver tissue, KEGG pathway enrichment analysis revealed that the identified lncRNAs were implicated in multiple metabolic pathways, including phenylalanine metabolism, linoleic acid metabolism, and retinol metabolism—key biochemical processes vital for hepatic functions (Figure 2E). Additionally, tissue-specific lncRNAs in other organs were enriched in pathways associated with their respective physiological functions/roles. In oocytes, KEGG enrichment analysis further indicated the involvement of lncRNAs in processes such as cell morphology regulation and developmental transitions (Figure 2F).

### 3.3. Analysis of Tissue-Specific lncRNA Expression Patterns Through WGCNA

**Figure 3:**
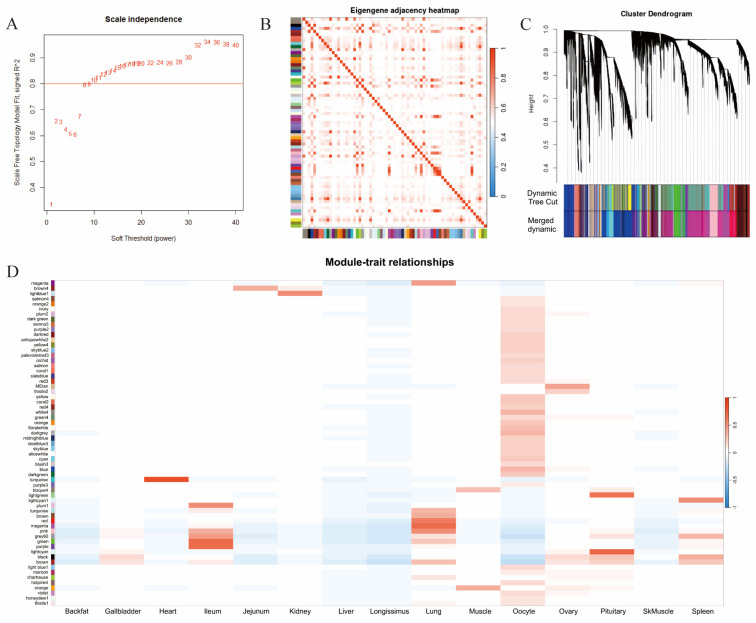
WGCNA of 15 pig tissues. (**A**) Selection of the soft-thresholding power (β) for scale-free topology model fit (R^2^ > 0.8). (**B**) Correlation heatmap illustrating the relationships between different gene modules. (**C**) WGCNA-based clustering dendrogram displaying lncRNA modules identified through dynamic tree cutting and subsequent module merging. Different branch colors represent distinct co-expression modules. (**D**) Heatmap depicting the correlation between the identified modules and various tissues; red indicates a positive correlation, while blue represents a negative correlation.

To enhance our understanding of the biological relationships and potential functions of core lncRNAs across various tissues, we conducted a Weighted Gene Co-expression Network Analysis (WGCNA) on lncRNA expression profiles. In this analysis, we determined an optimal soft-thresholding power (β) for constructing a scale-free network. A threshold with R^2^ > 0.8 was selected, and the final β value was set to 10 (Figure 3A).

To explore the differences in lncRNA expression patterns across different tissues, we performed t-SNE clustering analysis. The results revealed that seven out of the 15 tissues displayed distinct WGCNA-based clustering dendrograms (Figure 3B), including back fat, heart, jejunum, ileum, kidney, lung, and oocytes. This finding indicates that the expression patterns of lncRNAs in these tissues are highly tissue specific. Notably, the lncRNA expression profile in oocytes exhibited significant tissue specificity, suggesting that the genes and functions regulated by these lncRNAs may also be uniquely specialized (Figure 3B).

Through WGCNA-based clustering dendrograms clustering, followed by module refinement and merging, we identified a total of 35 modules (Figure 3C), each represented by a different color and corresponding to a group of lncRNAs with highly correlated expression patterns across tissues. Among these, six modules were significantly associated with tissue specificity (r > 0.65). For instance, the turquoise module showed a strong correlation with the heart (r = 0.85), the light cyan module was correlated with the pituitary (r = 0.75), and the magenta module correlated with the lung (r = 0.69; Figure 3D).

Furthermore, within these six tissue-specific modules, we identified hub lncRNAs based on module membership (MM) and gene significance (GS) metrics, applying thresholds of |GS| > 0.2 and |MM| > 0.8. This analysis resulted in the identification of 40, 429, 778, 1987, 184, and 125 hub lncRNAs in the heart–dark green, ileum–green, ileum–purple, lung–magenta, pituitary–light green, and pituitary–light cyan modules, respectively (Figure 3D). Although no module was specifically linked to oocytes, several modules demonstrated moderate correlations with oocyte samples, indicating that lncRNA expression patterns in oocytes may also possess tissue-specific characteristics.

### 3.4. Analysis of Conserved lncRNAs in Oocyte Development Across Species

**Figure 4:**
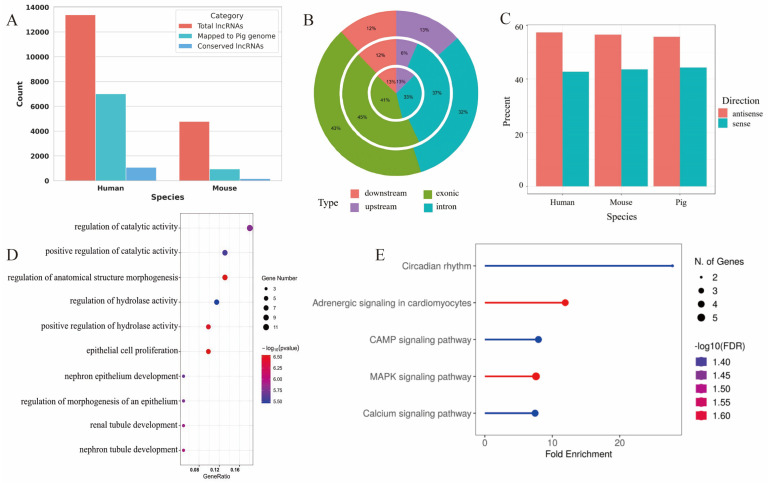
Analysis of conserved lncRNAs in oocyte development across species. (**A**) Summary of conserved lncRNA counts between pig and two other species (human and mouse). (**B**) Distribution of lncRNAs across genomic loci in pig, human, and mouse. (**C**) Distribution of strand orientation for conserved lncRNAs. (**D**) GO enrichment analysis of cis-regulatory target genes of human–pig conserved lncRNAs, highlighting their involvement in the regulation of catalytic activity, morphogenesis, and hydrolase activity. (**E**) KEGG pathway enrichment analysis of cis-regulatory target genes of mouse–pig conserved lncRNAs, revealing enrichment in pathways including cAMP, MAPK, and calcium signaling.

In this section, we focused on lncRNAs that may play critical roles in oocyte development, conducting a conservation analysis between pigs and both mice and humans. We hypothesize that these conserved lncRNAs are functionally significant in the regulation of oocyte development. In humans, we identified a total of 13,380 lncRNAs, of which 7013 were successfully aligned to the pig genome. Among these, 1078 lncRNAs exhibited positional homology with porcine lncRNAs and were classified as conserved, accounting for 8.1% of the total. Similarly, we identified 4782 lncRNAs in mice, with 950 aligning to the pig genome. Of these, 170 lncRNAs were found to be positionally conserved, representing 3.6% of the total (Figure 4A).

We then examined the genomic locations and transcriptional orientations of these conserved lncRNAs and found no significant differences compared to those of lncRNAs expressed in pig oocytes. These findings suggest that the conserved lncRNAs may exhibit oocyte-specific characteristics (Figure 4B,C).

To explore the potential functions of these conserved lncRNAs, we performed enrichment analysis on their predicted cis-regulatory target genes, specifically focusing on their roles in oocyte growth and development. GO enrichment analysis of the cis-regulatory target genes of human–pig conserved lncRNAs revealed significant enrichment in key biological processes, including the regulation of catalytic activity, anatomical structure morphogenesis, and hydrolase activity (Figure 4D).

Additionally, we conducted KEGG pathway analysis for the cis-regulatory target genes of mouse-pig conserved lncRNAs. The results indicated that these target genes were enriched in functional pathways such as the cAMP signaling pathway, mitogen-activated protein kinases (MAPK) signaling pathway, and calcium signaling pathway (Figure 4E). This analysis suggests that these signaling pathways may play regulatory roles during oocyte development.

### 3.5. Global Expression Patterns of lncRNAs During Follicular Development

**Figure 5:**
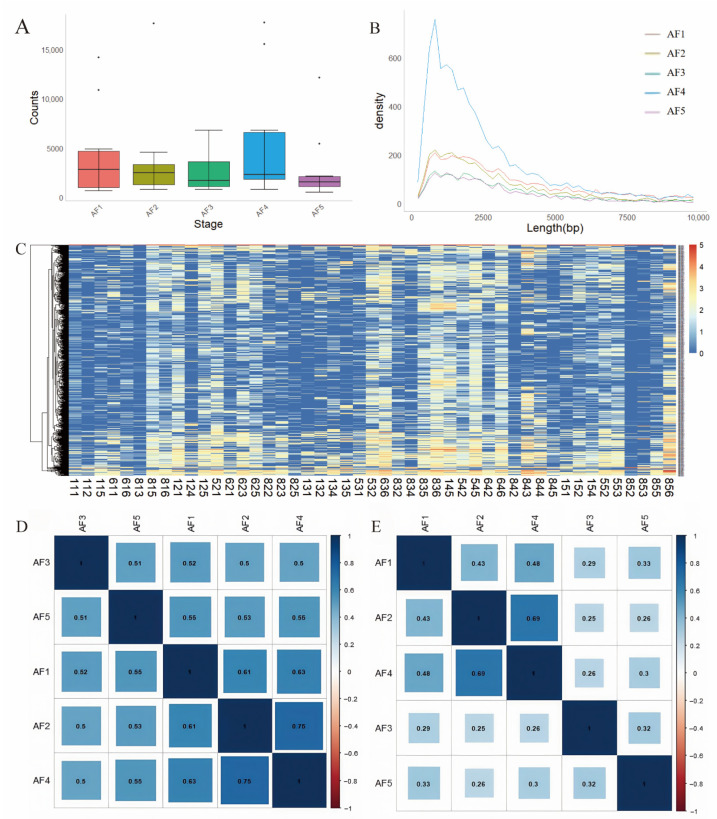
Expression patterns of lncRNAs during follicular development. (**A**) Summary of lncRNA counts across the five developmental stages of porcine oocytes. (**B**) Distribution of lncRNA lengths identified at each of the five oocyte stages. (**C**) Heatmap illustrating the expression levels of lncRNAs. In the sample names, the first digit indicates the individual, the second digit represents the developmental stage, and the third digit denotes the replicate number. (**D**) Correlation analysis of all expressed lncRNAs among the five developmental stages of porcine oocytes. (**E**) Correlation of tissue-specific lncRNAs across the five developmental stages.

To investigate the dynamic expression patterns of lncRNAs during follicular development, antral follicles were classified into five developmental stages based on their diameters: AF1 (0.5–1.8 mm), AF2 (1.8–3 mm), AF3 (3–5 mm), AF4 (5–7 mm), and AF5 (≥7 mm). Consistent with our multi-tissue analysis, we employed the same statistical approach to quantify the abundance of lncRNAs across these five stages. However, we observed no significant differences in lncRNA levels among the stages (Figure 5A). In line with previous findings, lncRNAs with lengths ranging from 800 to 900 nucleotides were the most prevalent (Figure 5B).

Additionally, we examined the expression profiles of lncRNAs in each sample and found no substantial variation across developmental stages, suggesting that lncRNA expression may be influenced by other regulatory factors (Figure 5C). To further explore the relationships in lncRNA expression across the five oocyte developmental stages, we performed Spearman correlation analysis. The results indicated relatively low correlation coefficients among the stages, suggesting limited similarity in the overall lncRNA expression profiles (Figure 5D,E). These findings indicate substantial differences in lncRNA expression profiles between developmental stages, suggesting that dynamic changes in lncRNA expression may play an important regulatory role during oocyte development.

### 3.6. Functional Analysis and Regulatory Networks of lncRNAs During Follicular Development

Both lncRNAs and circRNAs function as molecular sponges that competitively bind to miRNAs. In this study, we aimed to investigate the potential regulatory roles of lncRNAs and circRNAs on mRNAs by constructing a circRNA/lncRNA–miRNA–mRNA interaction network in porcine oocytes. This network was utilized to identify genes whose expression may be concurrently influenced by both types of non-coding RNAs.

**Figure 6:**
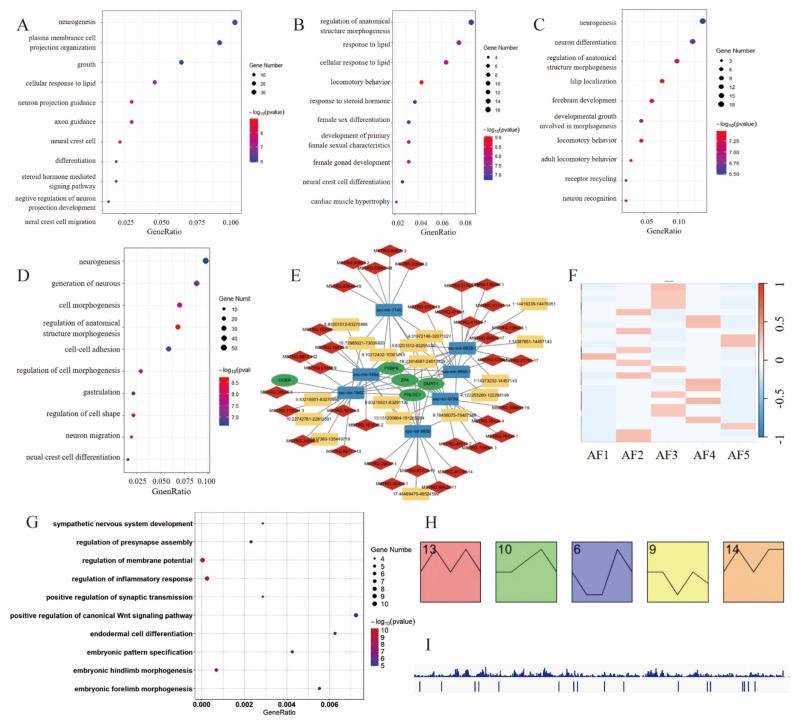
Characterization of tissue-specific lncRNAs and functional networks in porcine oocytes. (**A**) GO enrichment analysis of cis-regulated genes associated with differentially expressed lncRNAs between AF1 and AF2. (**B**) GO enrichment analysis between AF2 and AF3. (**C**) GO enrichment analysis between AF3 and AF4. (**D**) GO enrichment analysis between AF4 and AF5. (**E**) Tissue-specific ceRNA network of porcine oocytes. (**F**) WGCNA of lncRNAs expressed in oocytes. (**G**) GO enrichment analysis of mRNAs associated with key lncRNAs. (**H**) Expression patterns significantly enriched for oocyte lncRNAs. (**I**) Genomic loci of key lncRNAs: the top row represents chromosome length, the middle row shows positions of all lncRNAs, and the bottom row indicates positions of key lncRNAs.

To further investigate the expression dynamics of lncRNAs across the five developmental stages of porcine oocytes, we conducted a differential expression analysis among the stages and performed GO enrichment analysis of their predicted cis-regulatory target genes. Notably, the comparison between AF2 and AF3 revealed an enrichment of target genes in biological processes, including female sex differentiation, development of primary female sexual characteristics, and female gonad development. These findings suggest that the target genes of differentially expressed lncRNAs in AF2 and AF3 are enriched in sex differentiation–related pathways, indicating that these lncRNAs may play important roles in regulating oocyte maturation and follicle selection (Figure 6A–D).

We identified several miRNAs that may be co-regulated by both lncRNAs and circRNAs, along with their potential downstream target mRNAs. For instance, ssc−miR−1842 may be regulated by circRNAs such as circ−5:83215501–83270866 and lncRNAs like MSTRG.34546.5, potentially influencing the expression of oocyte expressed protein (OOEP). Similarly, ssc−miR−9855−1 may be regulated by circ−4:122253260–122298199 and MSTRG.76430.1, which might affect Zona pellucida glycoprotein 4 (ZP4 expression; Figure 6E). Comparable results were also observed in other tissues.

Following this, we performed WGCNA on the lncRNAs and identified 25 distinct modules. While some modules exhibited a positive correlation with specific developmental stages, the overall correlation was low, making it challenging to establish clear biological relationships between individual modules and specific stages (Figure 6F).

GO enrichment analysis of the cis-regulatory target genes of these key lncRNAs revealed significant associations with cellular morphological changes and cell differentiation processes (Figure 6G).

Using short time-series expression analysis, we classified the identified lncRNAs into 20 expression clusters to capture their temporal expression patterns. Among these, five clusters displayed markedly distinct expression profiles (Figure 6H), highlighting dynamic and potentially functional expression trajectories during oocyte development.

To elucidate the physiological roles of lncRNAs in oocytes, we screened for differentially expressed lncRNAs (Supplemental Tables S5 and S6) and selected those exhibiting five distinct expression patterns, which were designated as key lncRNAs (Figure 6I). This analysis led to the identification of 21 key lncRNAs (Table 1).

## 4. Discussion

In this study, we conducted a comprehensive analysis of lncRNAs across various porcine tissues and oocytes at different developmental stages. Our findings underscore the critical roles of lncRNAs in growth, development, and trait formation in pigs. We observed significant variations in lncRNA expression patterns among different tissues, with oocytes exhibiting the most distinct and pronounced lncRNA profiles. These results provide valuable theoretical insights into the regulatory mechanisms of lncRNAs, thereby deepening our understanding of porcine functional biology and development.

### 4.1. Expression Patterns of LncRNAs Across 15 Tissues

lncRNAs exhibit tissue-specific expression patterns, playing crucial roles in regulating gene expression, cellular differentiation, and development. Understanding lncRNAs profiles can provide insights into physiological functions, disease mechanisms, and potential therapeutic targets, enhancing precision medicine approaches for tissue-specific conditions [46,47].

lncRNAs are distributed throughout chromosomes, often situated near or within gene loci. While some lncRNAs exhibit tissue-specific expression, others play roles in regulating genes across different chromosomes [48]. Their precise locations can significantly influence their functions, including chromatin remodeling, transcriptional regulation, and interactions with other RNA molecules. In our study, we observed that lncRNAs were most abundant on Chromosome 1 and least abundant on the Y chromosome. Additionally, lncRNA density was highest on Chromosome 9 and lowest on the Y chromosome (Figure 1A,B).

Porcine Chromosome 1, one of the largest chromosomes in the pig genome, contains a substantial number of genes associated with various traits, including growth, reproduction, and immune response. Therefore, investigating gene functions and lncRNAs from this chromosome is vital for understanding their roles in economically important traits such as meat quality and disease resistance. Chromosome 9, although smaller than Chromosome 1, still harbors essential genetic information related to metabolic processes and growth regulation. Consequently, studying gene functions and lncRNAs from this chromosome will also provide valuable insights into traits such as fat deposition and carcass quality.

In contrast, the Y chromosome has a lower abundance of lncRNAs compared to the X chromosome and other chromosomes, primarily due to its smaller size and reduced gene content [49]. The Y chromosome is highly specialized and has undergone significant degeneration throughout evolutionary history, resulting in the loss of many genes and genetic material. This reduction limits the potential for lncRNA transcription on the Y chromosome.

The strand orientation of lncRNAs is essential for understanding their functions, as it can influence their interactions with other RNA molecules or proteins. In the study, we revealed that approximately 25% of lncRNAs were located on the sense strand, while 75% were found on the antisense strand (Figure 1E). Antisense lncRNAs can overlap with their target mRNAs, forming RNA:RNA complexes that can either stabilize or degrade the mRNA, depending on the context. For instance, an antisense lncRNA can have both overlapping and non-overlapping domains with its target mRNA, allowing for specific interactions and regulatory functions [50]. Sense lncRNAs can act as decoys or scaffolds for proteins, influencing gene expression by recruiting transcriptional regulators or modifying chromatin structure.

lncRNAs often possess multiple functional domains that facilitate interactions with proteins, other RNA molecules, and DNA, thereby playing crucial roles in various biological processes. In our study, we observed that the length distribution of lncRNAs is consistent across different tissues, with a peak length ranging from 800 to 900 nt. The longest lncRNA identified in this study exceeded 10,000 nt. Longer lncRNAs have the potential to adopt complex secondary and tertiary structures, which are vital for their stability and functionality [51]. The length of lncRNAs may contribute to the formation of these intricate structures, facilitating specific interactions with their molecular targets. The observed 800–900 nt range likely represents an optimal balance between providing sufficient length for effective interactions and maintaining the efficiency of transcription and processing [51].

In our study, we found that the majority of lncRNAs are located within exonic regions, with intronic regions being the second most common location. This distribution may be attributed to the fact that exonic lncRNAs often play more direct regulatory roles in relation to the protein-coding genes with which they are associated. In contrast, intronic lncRNAs may primarily influence the splicing and processing of pre-mRNA, thereby contributing to the regulation of gene expression at different levels [52].

Among all tissues analyzed, the ovary exhibited the lowest number of detected lncRNAs (2693), significantly fewer than other tissues. The reduced number of lncRNAs detected in oocytes may reflect their single-cell identity compared to heterogeneous tissues composed of multiple cell types. This feature highlights the transcriptional simplicity of oocytes at specific stages rather than a true reduction in regulatory complexity. Also, this may be related to the unique transcriptional state of oocytes, which are known to exist in either the surrounded nucleoli (SN) or non-surrounded nucleoli (NSN) state (3, 25). In contrast, the pituitary gland contained the highest number of lncRNAs (20,360). This discrepancy may be attributed to the fact that actively dividing or metabolically active cells require precise regulation of gene expression to support processes such as growth, differentiation, and responses to environmental signals. This heightened activity is associated with increased transcription of both protein-coding genes and lncRNAs [51].

### 4.2. Evolutionary Conservation of Oocyte lncRNAs Across Species

The evolutionary conservation of oocyte lncRNAs varies significantly across different species, reflecting the extent to which specific lncRNA sequences and their functions have been preserved throughout evolutionary history. This conservation is crucial, as sequences that remain intact over time often indicate essential roles in critical cellular processes [53].

While it is commonly understood that many lncRNAs experience weaker evolutionary constraints and exhibit lower levels of sequence conservation compared to protein-coding genes, a notable subset of lncRNAs demonstrates significant conservation across diverse species [54]. In many species, including mammals, birds, and amphibians, certain oocyte lncRNAs exhibit a high degree of sequence similarity, indicating their importance in oocyte development and maturation. For instance, lncRNAs that regulate key genes involved in oocyte meiosis and folliculogenesis are often conserved, highlighting their critical roles in reproductive success [55].

In our investigation of oocyte lncRNAs, we identified 1078 conserved lncRNAs, of which 810 displayed pig-specific or pig-preferred expression patterns, while 268 showed a preference for expression in humans. The conservation of lncRNAs suggests they may play vital roles in fundamental biological processes, including development, cell differentiation, and responses to environmental changes. The loss of such conserved lncRNAs could potentially have detrimental effects on organismal fitness, highlighting their significance in evolutionary biology [53].

To further explore the potential functions of lncRNAs, we categorized housekeeping lncRNAs based on their genomic positioning and strand orientation. GO and KEGG enrichment analyses revealed that lncRNAs located in intronic regions and on the antisense strand are particularly enriched in pathways related to gene expression regulation, epigenetic modification, and embryonic development. These findings are consistent with previous research demonstrating that intronic lncRNAs play a vital role in regulating gene expression, alternative splicing, and RNA processing [54]. Intronic lncRNAs influence transcriptional interference, maintain nuclear architecture, function as ceRNAs, and modulate epigenetic modifications, thereby contributing significantly to developmental processes and cellular functions. Similarly, lncRNAs located on the antisense strand regulate gene expression by modulating transcription, influencing alternative splicing, and recruiting chromatin modifiers. They can also act as ceRNAs, sequestering microRNAs and facilitating the nuclear retention of mRNAs, which ultimately impacts various cellular functions [56].

### 4.3. Oocyte Development Stages

Oocytes exhibit the lowest levels of lncRNA expression among the analyzed tissues, primarily due to their specific developmental stage, streamlined regulatory mechanisms, and focus on essential functions necessary for fertilization and early embryonic development. This distinctive transcriptional landscape prioritizes critical processes, leading to reduced lncRNA activity compared to other tissues [57,58]. In our study, we observed no significant differences in lncRNA levels across the developmental stages, likely for the reasons mentioned above.

Using the Spearman correlation analysis, we found limited similarity in lncRNA expression profiles across the five developmental stages of oocyte maturation. These findings suggest that dynamic changes in lncRNA expression are essential for the proper regulation of oocyte development. The variations in lncRNA expression may be linked to specific developmental processes, signaling pathways, or cellular functions unique to each stage, underscoring the importance of lncRNAs in facilitating transitions between developmental phases and ensuring the successful maturation and functionality of oocytes [56].

To investigate the expression dynamics of lncRNAs across the five developmental stages of porcine oocytes, we conducted a differential expression analysis among 5 stages and performed GO enrichment analysis of their predicted cis-regulatory target genes. The comparison between the AF2 (1.8–3 mm) and AF3 (3–5 mm) stages revealed significant enrichment of target genes associated with key biological processes, including female sex differentiation, the development of primary female sexual characteristics, and female gonad development. During the AF2 stage, follicles exhibit increased responsiveness to hormonal signals, particularly follicle-stimulating hormone (FSH). In pigs, follicles transition from the secondary follicle to the antral follicle when their diameter is less than 3 mm [3]. At this stage, the oocyte is classified as a growing oocyte and does not resume meiosis in vitro. This period is crucial for selecting which follicles will continue to develop. In contrast, during the AF3 stage, the oocyte within the follicle is actively growing and maturing. Oocytes achieve full meiotic competence when their diameter reaches ≥3 mm, signifying that they have attained cytoplasmic maturity [3].

### 4.4. Limitations of Our Study

Our study has several limitations. First, while we analyzed a broad range of porcine tissues, the primary focus was on 14 specific tissues and oocytes. Including additional tissue types, especially those involved in reproductive and developmental processes, could provide deeper insights into the functions of lncRNAs. Second, although our analysis incorporated a substantial number of RNA-seq datasets, the variability in sample sizes among different tissues may affect the robustness of our findings. Third, the conservation analysis of lncRNAs across species, specifically humans and mice, may be constrained by differences in evolutionary pressures and the functional roles of lncRNAs in various species. As a result, our findings may not fully capture the functional significance of conserved lncRNAs in pigs. Finally, the study primarily focused on differential expression patterns without thoroughly investigating the mechanistic roles of specific lncRNAs in regulating target genes or biological processes. Conducting functional validation of selected lncRNAs in spleen tissue would enhance the conclusions drawn from this research.

## 5. Conclusions

Our research on lncRNAs across various porcine tissues enhances the understanding of their diverse roles and tissue-specific functions, with significant implications for animal production and health/disease. Further exploration of lncRNAs may lead to improved reproductive efficiency and deeper insights into developmental biology in pigs and other mammals. Ultimately, these analyses underscore the involvement of lncRNAs in critical biological processes, particularly during oocyte development, and highlight their potential for informing the development of new biomarkers and therapeutic strategies.

## Figures and Tables

**Table 1:** 21 Key lncRNAs for oocyte development.

LncRNAs ID	Profile ID
MSTRG.43628.73	6
MSTRG.115456.4	10
MSTRG.44539.14	10
MSTRG.2129.22	13
MSTRG.77677.3	13
MSTRG.81716.7	13
MSTRG.56521.8	13
MSTRG.27723.2	13
MSTRG.55615.19	13
MSTRG.60376.11	13
MSTRG.70292.28	13
MSTRG.33636.10	13
MSTRG.16120.5	13
MSTRG.43002.6	13
MSTRG.84430.5	13
MSTRG.106359.2	13
MSTRG.10345.3	13
MSTRG.109031.13	13
MSTRG.117183.1	13
MSTRG.68892.3	13
MSTRG.121408.16	13
MSTRG.76430.4	13
MSTRG.100322.23	13
MSTRG.54358.12	13
MSTRG.105702.44	14

## Data Availability

The data presented in this study are available in this article (Table S1).

## Supplementary Materials

The following supporting information can be downloaded at: https://www.mdpi.com/article/10.3390/cells14181409/s1. Table S1: Summary of SRR accession numbers for the 607 RNA-Seq datasets. Table S2: Sequences and genomic locations of the selected housekeeping lncRNAs used for validation; Table S3: PCR reaction mixture composition for the validation of housekeeping lncRNAs; Table S4: PCR thermal cycling conditions applied for the amplification of housekeeping lncRNAs; Table S5: Genes highly correlated with the continuous upregulation of lncRNAs; Table S6: Genes highly correlated with the continuous down-regulation of lncRNAs.

## Abbreviations

LncRNA	Long Non-coding RNA
UTR	Untranslated Region
mRNA	Manager RNA
miRNA	Micro RNA
circRNA	Circular RNA
RNA-seq	RNA sequencing
ORF	Open Reading Frame
GV	Germinal vesicle
NCBI	National Central of Biotechnology Information
t-SNE	t-distributed stochastic neighbor embedding
CeRNA	competing endogenous RNA
GO	Gene Ontology
KEGG	Kyoto Encyclopedia of Genes and Genomes
WGCNA	Weighted Gene Co-Expression Network Analysis
PCR	Polymerase Chain Reaction
SN	Surrounded nucleolus
NSN	Non surrounded nucleolus

## References

[1] Maes D.G.D., Dewulf J., Piñeiro C., Edwards S., Kyriazakis I. (2020). A critical reflection on intensive pork production with an emphasis on animal health and welfare. J. Anim. Sci..

[2] Zhao S., Guo Z., Xiang W., Wang P. (2021). The neuroendocrine pathways and mechanisms for the control of the reproduction in female pigs. Anim. Reprod..

[3] Jiang Y., He Y., Pan X., Wang P., Yuan X., Ma B. (2023). Advances in Oocyte Maturation In Vivo and In Vitro in Mammals. Int. J. Mol. Sci..

[4] Whitworth K.M., Green J.A., Redel B.K., Geisert R.D., Lee K., Telugu B.P., Wells K.D., Prather R.S. (2022). Improvements in pig agriculture through gene editing. CABI Agric. Biosci..

[5] Hou N., Du X., Wu S. (2022). Advances in pig models of human diseases. Anim. Models Exp. Med..

[6] Meyerholz D.K., Burrough E.R., Kirchhof N., Anderson D.J., Helke K.L. (2024). Swine models in translational research and medicine. Vet. Pathol..

[7] Xu C., Fang X., Xu X., Wei X. (2024). Genetic engineering drives the breakthrough of pig models in liver disease research. Liver Res..

[8] Mattick J.S., Amaral P.P., Carninci P., Carpenter S., Chang H.Y., Chen L.-L., Chen R., Dean C., Dinger M.E., Fitzgerald K.A. (2023). Long non-coding RNAs: Definitions, functions, challenges and recommendations. Nat. Rev. Mol. Cell Biol..

[9] Chowdhary A., Satagopam V., Schneider R. (2021). Long Non-coding RNAs: Mechanisms, Experimental, and Computational Approaches in Identification, Characterization, and Their Biomarker Potential in Cancer. Front. Genet..

[10] Statello L., Guo C.-J., Chen L.-L., Huarte M. (2021). Gene regulation by long non-coding RNAs and its biological functions. Nat. Rev. Mol. Cell Biol..

[11] Aliperti V., Skonieczna J., Cerase A. (2021). Long Non-Coding RNA (lncRNA) Roles in Cell Biology, Neurodevelopment and Neurological Disorders. Non-Coding RNA.

[12] Coan M., Haefliger S., Ounzain S., Johnson R. (2024). Targeting and engineering long non-coding RNAs for cancer therapy. Nat. Rev. Genet..

[13] Badowski C., He B., Garmire L.X. (2022). Blood-derived lncRNAs as biomarkers for cancer diagnosis: The Good, the Bad and the Beauty. npj Precis. Oncol..

[14] Guh C.-Y., Hsieh Y.-H., Chu H.-P. (2020). Functions and properties of nuclear lncRNAs-from systematically mapping the interactomes of lncRNAs. J. Biomed. Sci..

[15] Samaddar S., Banerjee S. (2021). Far from the nuclear crowd: Cytoplasmic lncRNA and their implications in synaptic plasticity and memory. Neurobiol. Learn. Mem..

[16] Tan Y.-T., Lin J.-F., Li T., Li J.-J., Xu R.-H., Ju H.-Q. (2021). LncRNA-mediated posttranslational modifications and reprogramming of energy metabolism in cancer. Cancer Commun..

[17] Wu L., Li L., Liang Y., Chen X., Mou P., Liu G., Sun X., Qin B., Zhang S., Zhao C. (2021). Identification of differentially expressed long non-coding RNAs and mRNAs in orbital adipose/connective tissue of thyroid-associated ophthalmopathy. Genomics.

[18] Jiang C., Li Y., Zhao Z., Lu J., Chen H., Ding N., Wang G., Xu J., Li X. (2016). Identifying and functionally characterizing tissue-specific and ubiquitously expressed human lncRNAs. Oncotarget.

[19] Srinivas T., Mathias C., Oliveira-Mateos C., Guil S. (2023). Roles of lncRNAs in brain development and pathogenesis: Emerging therapeutic opportunities. Mol. Ther. J. Am. Soc. Gene Ther..

[20] Wang S., Jin J., Xu Z., Zuo B. (2019). Functions and Regulatory Mechanisms of lncRNAs in Skeletal Myogenesis, Muscle Disease and Meat Production. Cells.

[21] Ma L., Qin M., Zhang Y., Xue H., Li S., Chen W., Zeng Y. (2022). Identification and functional prediction of long non-coding RNAs related to skeletal muscle development in Duroc pigs. Anim. Biosci..

[22] Yu L., Tai L., Gao J., Sun M., Liu S., Huang T., Yu J., Zhang Z., Miao W., Li Y. (2022). A New lncRNA, lnc-LLMA, Regulates Lipid Metabolism in Pig Hepatocytes. DNA Cell Biol..

[23] Jin L., Tang Q., Hu S., Chen Z., Zhou X., Zeng B., Wang Y., He M., Li Y., Gui L. (2021). A pig BodyMap transcriptome reveals diverse tissue physiologies and evolutionary dynamics of transcription. Nat. Commun..

[24] Gong Y., He J., Li B., Xiao Y., Zeng Q., Xu K., Duan Y., He J., Ma H. (2021). Integrated Analysis of lncRNA and mRNA in Subcutaneous Adipose Tissue of Ningxiang Pig. Biology.

[25] Yuan X., Chen N., Feng Y., Li N., Pan X., Tian Y., Wang J., Jiang Y., He D., Li J. (2023). Single-cell multi-omics profiling reveals key regulatory mechanisms that poise germinal vesicle oocytes for maturation in pigs. Cell. Mol. Life Sci..

[26] Aalto A.P., Pasquinelli A.E. (2012). Small non-coding RNAs mount a silent revolution in gene expression. Curr. Opin. Cell Biol..

[27] Friedman R.C., Farh K.K.-H., Burge C.B., Bartel D.P. (2009). Most mammalian mRNAs are conserved targets of microRNAs. Genome Res..

[28] Huang W., Xiong T., Zhao Y., Heng J., Han G., Wang P., Zhao Z., Shi M., Li J., Wang J. (2024). Computational prediction and experimental validation identify functionally conserved lncRNAs from zebrafish to human. Nat. Genet..

[29] Dhanoa J.K., Sethi R.S., Verma R., Arora J.S., Mukhopadhyay C.S. (2018). Long non-coding RNA: Its evolutionary relics and biological implications in mammals: A review. J. Anim. Sci. Technol..

[30] Tsoi S., Zhou C., Grant J.R., Pasternak J., Dobrinsky J., Rigault P., Nieminen J., Sirard M.-A., Robert C., Foxcroft G.R. (2012). Development of a porcine (Sus scofa) embryo-specific microarray: Array annotation and validation. BMC Genom..

[31] Chen S., Zhou Y., Chen Y., Gu J. (2018). Fastp: An ultra-fast all-in-one FASTQ preprocessor. Bioinformatics.

[32] Pertea M., Pertea G.M., Antonescu C.M., Chang T.-C., Mendell J.T., Salzberg S.L. (2015). StringTie enables improved reconstruction of a transcriptome from RNA-seq reads. Nat. Biotechnol..

[33] Pertea G., Pertea M. (2020). GFF Utilities: GffRead and GffCompare. F1000Research.

[34] Sun L., Luo H., Bu D., Zhao G., Yu K., Zhang C., Liu Y., Chen R., Zhao Y. (2013). Utilizing sequence intrinsic composition to classify protein-coding and long non-coding transcripts. Nucleic Acids Res..

[35] Kang Y.-J., Yang D.-C., Kong L., Hou M., Meng Y.-Q., Wei L., Gao G. (2017). CPC2: A fast and accurate coding potential calculator based on sequence intrinsic features. Nucleic Acids Res..

[36] Liao Y., Smyth G.K., Shi W. (2014). featureCounts: An efficient general purpose program for assigning sequence reads to genomic features. Bioinformatics.

[37] Kobak D., Berens P. (2019). The art of using t-SNE for single-cell transcriptomics. Nat. Commun..

[38] Spearman C. (2010). The proof and measurement of association between two things. Int. J. Epidemiol..

[39] She X., Rohl C.A., Castle J.C., Kulkarni A.V., Johnson J.M., Chen R. (2009). Definition, conservation and epigenetics of housekeeping and tissue-enriched genes. BMC Genom..

[40] Gao Y., Wang J., Zhao F. (2015). CIRI: An efficient and unbiased algorithm for de novo circular RNA identification. Genome Biol..

[41] Peng C., Wang S., Yu J., Deng X., Ye H., Chen Z., Yao H., Cai H., Li Y., Yuan Y. (2022). lncRNA-mRNA Expression Patterns in Invasive Pituitary Adenomas: A Microarray Analysis. BioMed Res. Int..

[42] Shannon P., Markiel A., Ozier O., Baliga N.S., Wang J.T., Ramage D., Amin N., Schwikowski B., Ideker T. (2003). Cytoscape: A software environment for integrated models of biomolecular interaction networks. Genome Res..

[43] Ge S.X., Jung D., Yao R. (2020). ShinyGO: A graphical gene-set enrichment tool for animals and plants. Bioinformatics.

[44] Langfelder P., Horvath S. (2008). WGCNA: An R package for weighted correlation network analysis. BMC Bioinform..

[45] Lee B.T., Barber G.P., Benet-Pagès A., Casper J., Clawson H., Diekhans M., Fischer C., Gonzalez J.N., Hinrichs A.S., Lee C.M. (2022). The UCSC Genome Browser database: 2022 update. Nucleic Acids Res..

[46] Marttila S., Chatsirisupachai K., Palmer D., de Magalhães J.P. (2020). Ageing-associated changes in the expression of lncRNAs in human tissues reflect a transcriptional modulation in ageing pathways. Mech. Ageing Dev..

[47] Chen X., Sun Z. (2021). Novel lincRNA Discovery and Tissue-Specific Gene Expression across 30 Normal Human Tissues. Genes.

[48] Wang W., Min L., Qiu X., Wu X., Liu C., Ma J., Zhang D., Zhu L. (2021). Biological Function of Long Non-coding RNA (LncRNA) Xist. Front. Cell Dev. Biol..

[49] Skinner B.M., Sargent C.A., Churcher C., Hunt T., Herrero J., Loveland J.E., Dunn M., Louzada S., Fu B., Chow W. (2016). The pig X and Y Chromosomes: Structure, sequence, and evolution. Genome Res..

[50] Ernst C., Morton C.C. (2013). Identification and function of long non-coding RNA. Front. Cell. Neurosci..

[51] Wang K.C., Chang H.Y. (2011). Molecular mechanisms of long noncoding RNAs. Mol. Cell.

[52] Chodurska B., Kunej T. (2025). Long non-coding RNAs in humans: Classification, genomic organization and function. Non-Coding RNA Res..

[53] Tsagakis I., Douka K., Birds I., Aspden J.L. (2020). Long non-coding RNAs in development and disease: Conservation to mechanisms. J. Pathol..

[54] Ramírez-Colmenero A., Oktaba K., Fernandez-Valverde S.L. (2020). Evolution of Genome-Organizing Long Non-coding RNAs in Metazoans. Front. Genet..

[55] Wang P., Paquet É.R., Robert C. (2023). Comprehensive transcriptomic analysis of long non-coding RNAs in bovine ovarian follicles and early embryos. PLoS ONE.

[56] Zhou M., Guo X., Wang M., Qin R. (2021). The patterns of antisense long non-coding RNAs regulating corresponding sense genes in human cancers. J. Cancer.

[57] Wang L., Li B., Cheng D. (2024). Influence of Long Non-Coding RNAs on Human Oocyte Development. Pharmacogenom. Pers. Med..

[58] He C., Wang K., Gao Y., Wang C., Li L., Liao Y., Hu K., Liang M. (2021). Roles of Noncoding RNA in Reproduction. Front. Genet..

